# *Heliocephala variabilis* and *Pseudopenidiella vietnamensis*: Two New Hyphomycetous Species in the Microthyriaceae (Dothideomycetes) from Vietnam

**DOI:** 10.3390/microorganisms8040478

**Published:** 2020-03-27

**Authors:** Isabel Iturrieta-González, Dania García, Josep Guarro, Josepa Gené

**Affiliations:** Unitat de Micologia, Facultat de Medicina i Ciències de la Salut and IISPV, Universitat Rovira i Virgili, Reus, 43201 Tarragona, Spain; isabeliturrieta@gmail.com (I.I.-G.); josep.guarro@urv.cat (J.G.); josepa.gene@urv.cat (J.G.)

**Keywords:** hyphomycetes, dematiaceous fungi, phylogeny, taxonomy, Vietnam

## Abstract

In a survey of microfungi from plant debris collected in Vietnam, two new hyphomycetous species were found, which belong to the genera *Heliocephala* and *Pseudopenidiella* and the family *Microthyriaceae* (*Microthyriales*, *Dothideomycetes*). Maximum Likelihood and Bayesian Inference sequence analyses of the internal transcribed spacers (ITS) and large subunit (LSU) of the ribosomal DNA barcodes allowed assessing the phylogenetic relationships of the new species with other species of the respective genera. *Heliocephala* *variabilis* sp. nov. was closely related to *Heliocephala elegans*, *Heliocephala gracilis*, and *Heliocephala zimbabweensis*, from which it was morphologically distinguished by its smaller conidiophores and non-rostrate conidia of up to four septa on the natural substratum. *Pseudopenidiella vietnamensis* sp. nov. was related to *Pseudopenidiella piceae* and *Pseudopenidiella podocarpi* and differed from the former principally by its lack of microcondiophores and from *P. podocarpi* by having larger macroconidiophores and smooth conidia. Key morphological features to distinguish the accepted species in *Heliocephala* and *Pseudopenidiella* are also provided. In addition, *Pseudopenidiella pini* was excluded from the genus on the basis of its morphological features.

## 1. Introduction

Vietnam is one of the twenty most bio-diverse countries in the world [1,2,3]. Its very diverse ecological niches, climatic conditions, and high level of plant endemism would suggest that the country also has great fungal diversity, although this has been not extensively studied so far [4,5,6]. During a survey of asexual microfungi from plant debris carried out in the Northeast of Vietnam, two interesting specimens, belonging to the genera *Heliocephala* and *Pseudopenidiella* (*Microthyriaceae*, *Microthyriales, Dothideomycetes*) [7] were isolated and deposited in the culture collection at the Medicine Faculty of the Universitat Rovira i Virgili (Reus, Spain) for subsequent studies.

The genus *Heliocephala* was introduced by Rao et al. [8] based on *Heliocephala proliferans,* a hyphomycetous fungus characterized by the production of solitary macronematous conidiophores, bearing a terminal compact clusters of monoblastic conidiogenous cells that can arise more or less radially from short branches (metula-like) and produce obclavate, rostrate, or hooked conidia. Based on DNA data and morphological features, the genus was emended by Heredia-Abarca et al. [9] to include the species of *Holubovaniella* [10], the latter subsequently being considered a synonym of *Heliocephala*. Currently, *Heliocephala* comprises seven species, most of them isolated from plant material, with the exception of *Heliocephala natarajanii*, which was found along with a basidiocarp of *Pisolithus tinctorius* [11].

Crous et al. [12] introduced the genus *Pseudopenidiella* to accommodate *Pseudopenidiella piceae* recovered from the needle litter of *Picea abies* in the Czech Republic. Morphologically, the genus is characterized by the presence of micro- and macroconidiophores, with aseptate conidia and ramoconidia arranged in branched acropetal chains. It shows a conidiogenous apparatus similar to those of the genera *Cladosporium*, *Digitopodium*, and *Penidiella*, but mainly differs from them by the lack of darkened and coronate-type scars in both conidia and conidiogenous cells and by the absence of rhizoids associated with the conidiophores. Phylogenetically, three species have been recognized in the genus, *Pseudopenidiella podocarpi* being the most recently described on leaves of *Podocarpus latifolus,* collected in South Africa [13].

Based on a polyphasic approach, we propose two novel hyphomycetous fungi from Vietnam, *Heliocephala variabilis* and *Pseudopenidiella vietnamensis*, which are described and illustrated below.

## 2. Material and Methods

### 2.1. Samples and Isolates

Samples of plant debris were collected in the Northeast region of Vietnam in 2011 and treated according to Hernández-Restrepo et al. [14]. They were placed in moist chambers and incubated at room temperature (22 °C) in darkness, being examined periodically under a stereomicroscope over a 3-month period. The fungi were isolated on potato dextrose agar (PDA; Pronadisa, Madrid, Spain), and the interesting specimens were preserved at room temperature on PDA slant cultures covered with mineral oil.

### 2.2. DNA Extraction, PCR, Sequencing and Phylogenetic Analyses

Isolates were grown on PDA for 14 days at 25 °C in darkness, and DNA was extracted from mycelium through the modified protocol of Müller et al. [15]. For identification purposes, we performed the PCR using the primer pairs ITS5/ITS4 and NL1/NL4b to amplify the region spanning the internal transcribed spacers (ITS) 1 and 2, including the 5.8S gene, and the D1/D2 domain of the large subunit (LSU) of the nuclear ribosomal (nr)DNA, respectively, following the protocol of Cano et al. [16]. The PCR products were purified and stored at −20 °C until sequencing at Macrogen (Madrid, Spain).

ITS and LSU sequences of the unidentified isolates were compared with those of other fungi deposited at the National Center for Biotechnology Information (NCBI) by the Basic Local Alignment Search Tool (BLAST). To assess the taxonomic position and phylogenetic relationships of our fungi, we carried out single analyses of the ITS and LSU sequences obtained here and those available of *Heliocephala* and *Pseudopenidiella* species and other members of the family *Microthyriaceae*. Since a similar topology of the phylogenetic trees obtained from the previous analyses was observed, a combined analysis of the two markers was performed to obtain a more robust phylogeny of the fungi studied. All the sequences, which were mainly taken from previous studies [7,9,12,13,17,18,19,20], were retrieved from GenBank, including those of *Venturia catenospora* and *Venturia inaequalis* used as outgroup (Table 1). The alignments were made in the MEGA (Molecular Evolutionary Genetics Analysis) software v.6.0. [21], using the ClustalW algorithm [22] and refined with MUSCLE [23] or manually if necessary, on the same platform. Phylogenetic reconstructions were made using Maximum Likelihood (ML) and Bayesian Inference (BI) approaches using the MEGA software v. 6.0. [21] and MrBayes v. 3.2.6 [24], respectively.

For the ML analysis of the ITS and LSU regions, the best nucleotide substitution model determined by the same program was the Tamura–Nei model with Gamma distribution and invariant sites (T93+G+I); ML bootstrap values (BML) ≥ 70 % were considered significant. For the BI analysis, the best nucleotide substitution model was determined using jModelTest [25]. For the ITS region, we used the Kimura 2-parameter with Gamma distribution (K80+G) and for the LSU region, we used General Time Reversible with Gamma distribution and invariant sites (GTR+G+I). The parameters used were two simultaneous runs of 5,000,000 generations, four Markov chains, sampled every 1000 generations. The 50% majority-rule consensus tree and posterior probability values (PP) were calculated after discarding the first 25% of the samples. A PP value ≥ 0.95 was considered significant.

Sequences of the fungi studied here were deposited in GenBank (Table 1), and the alignment used was deposited in TreeBASE (submission number S25760).

### 2.3. Phenotypic Study

Microscopic characterization of the isolates was carried out on potato carrot agar (PCA; potato 20 g, carrot 20 g, agar 13 g, distilled water 1 L) and oatmeal agar (OA; Oatmeal 30 g, agar 13 g, distilled water 1 L) after 30 days of incubation to get sporulation. Measurements and descriptions of the structures were taken from the specimens mounted in Shear’s solution or lactic acid (100% *v*/*v*). Photomicrographs were obtained using a Zeiss Axio-Imager M1 light microscope (Zeiss, Oberkochen, Germany) with a DeltaPix Infinity X digital camera.

Macroscopic characterization of the colonies was done on PDA, OA, and PCA after 30 days of incubation at 25 °C in darkness. Cardinal temperatures for growth were obtained on PDA incubated at 5, 15, 20, 25, 30, 35, 37, and 40 °C after 14 days in darkness. The colony colors in descriptions are based on Kornerup and Wanscher [26]. 

Nomenclatural novelties and descriptions were deposited in MycoBank [27]. Ex-type cultures and holotypes, which were dried cultures, were deposited at the Westerdijk Fungal Biodiversity Institute (CBS, Utrecht, The Netherlands).

## 3. Results

**Figure 1:**
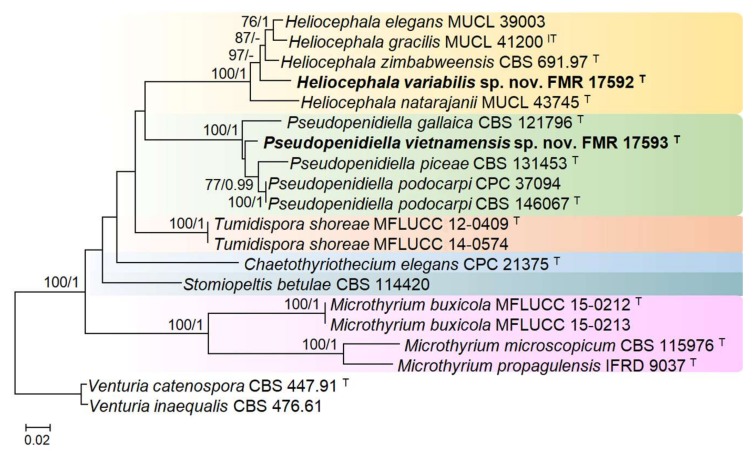
Maximum Likelihood (ML) tree constructed with the internal transcribed spacers (ITS) and large subunit (LSU) sequences of 18 strains representative of the family *Microthyriaceae* (*Microthyriales*). The phylogenetic tree was rooted with *V. catenospora* and *V. inaequalis* (*Venturiaceae*, *Venturiales*). Bootstrap support values for ML greater than 70% and Bayesian posterior probabilities greater than 0.95 are given near nodes. The names of the newly described species are in bold. Branch lengths are proportional to distance; ^T^ Ex-type strain; ^IT^ Ex-isotype strain.

BLAST analyses with LSU and ITS sequences confirmed the morphological identification of FMR 17592 and FMR 17593 at the genus level but revealed a relatively low percentage of identity with respect to other species, suggesting they were novel species of *Heliocephala* and *Pseudopenidiella*, respectively. The similarity of LSU sequences between FMR 17592 and other *Heliocephala* species (i.e., *Heliocephala gracilis*, *Heliocephala zimbabweensis*, *Heliocephala elegans*, and *H. natarajanii*) ranged from 94.04% to 96.73%. In the case of FMR 17593, similarity was 97.68% to *P. podocarpi*, 96.24% to *P. piceae*, and 95.80% to *Pseudopenidiella gallaica*. ITS sequences showed lower percentages of identity, with a maximum of 95.10% between FMR 17592 and the *Heliocephala* species mentioned above and of ≤89.25% between FMR 17593 and the species of *Pseudopenidiella* analyzed. A combined analysis of the two loci (ITS and LSU) revealed the status of these fungi with respect to the other species of *Heliocephala* and *Pseudopenidiella* and allied genera of the family *Microthyriaceae* (Figure 1). The total alignment included 20 sequences and comprised 1501 bp, from which 604 bp were variable, 864 bp conserved, and 460 bp phylogenetically informative. FMR 17592 and FMR 17593 were included in the full-supported clades representatives of the genera above mentioned and were genetically and morphologically differentiated from their closest phylogenetic relatives.

Key morphological features that distinguish the accepted species of *Heliocephala* and *Pseudopenidiella*, including the new taxa described below, are summarized in Table 2 and Table 3.

### Taxonomy

**Figure 2:**
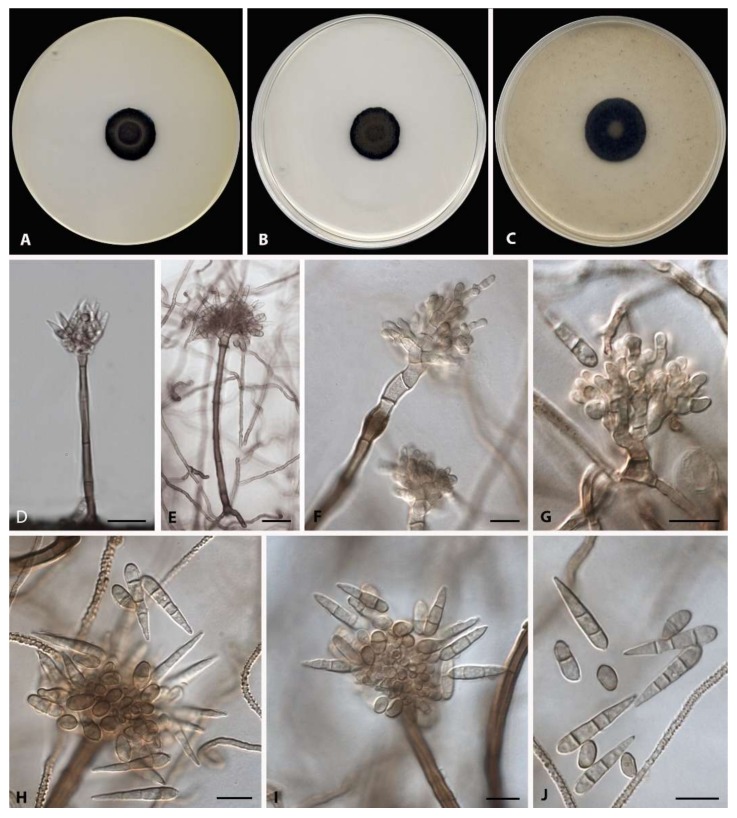
*H. variabilis* (ex-type FMR 17592). (**A**–**C**). Colonies on potato dextrose agar (PDA), potato carrot agar (PCA), and oatmeal agar (OA), respectively, after 30 days at 25 °C. (**D**–**J**). Conidiophores and conidia. Scale bars: (**D**,**E**) = 20 µm; (**F**–**J**) = 10 µm.

*Heliocephala variabilis* Iturrieta-González, Gené, Dania García, sp. nov.—MycoBank MB 833179 (Figure 2).

Etymology: Name refers to the variation in the conidial morphology.

Mycelium consisting of branched, septate, subhyaline to pale brown, smooth to verrucose hyphae 1–2 µm wide. Conidiophores macronematous, rarely semi-macronematous, mononematous, erect, subcylindrical, with up to seven septa, brown, pale brown towards the apex, smooth-walled, up to 153 µm long (up to 148 µm long on the natural substratum), 4–6 µm wide, commonly 2–3 closely packed primary branched, from which 1–2 secondary metula-like branches are commonly present. Conidiogenous cells terminal, monoblastic, discrete, ampuliform, smooth-walled, pale brown, 3–13 × 2–3 µm. Conidia solitary, broadly ellipsoidal, subcylindrical or obclavate, smooth-walled to slightly verruculose, pale brown, (0–)1–3-septate (up to 4-septate on the natural substratum): 0–1-septate, 4–11 × 3–6 µm; 2–3-septate 15–26 × 3–5 µm; 4-septate 24–26 × 3.8–4.3 µm. Sexual morph not observed.

Culture characteristics after 30 days at 25 °C—Colonies on PDA reaching 17–18 mm of diameter, olive color (3F7) with some areas olive-brown (4E4), velvety, convex, aerial mycelium scarce, margin slightly irregular; reverse dark brown (7F8) to black. On PCA, reaching 18 mm of diameter, yellowish brown (5E4), black at the periphery, velvety, flat, aerial mycelium scarce, margin slightly irregular; reverse dark brown (7F8) to black. On OA, reaching 23 mm of diameter, yellowish brown (5E4), black at the periphery, umbonate, slightly floccose at the center, velvety towards the periphery, margin entire and slightly fimbriate; reverse dark brown (7F8) to black.

Cardinal temperatures for growth: Minimum 15 °C, optimum 25 °C, maximum 30 °C.

Specimen examined: Vietnam, north-east region, on unidentified dead leaf, Aug. 2011, Josep Guarro (holotype CBS H-24291, culture ex-type CBS 146334 = FMR 17592).

Diagnosis: *H. variabilis* differs from four closely related species, i.e., *H. elegans*, *H. gracilis*, *H. zimbabweensis*, and *H. natarajanii*, by the size of its macronematous conidiophores and the septation of conidia (Table 2). 

**Figure 3:**
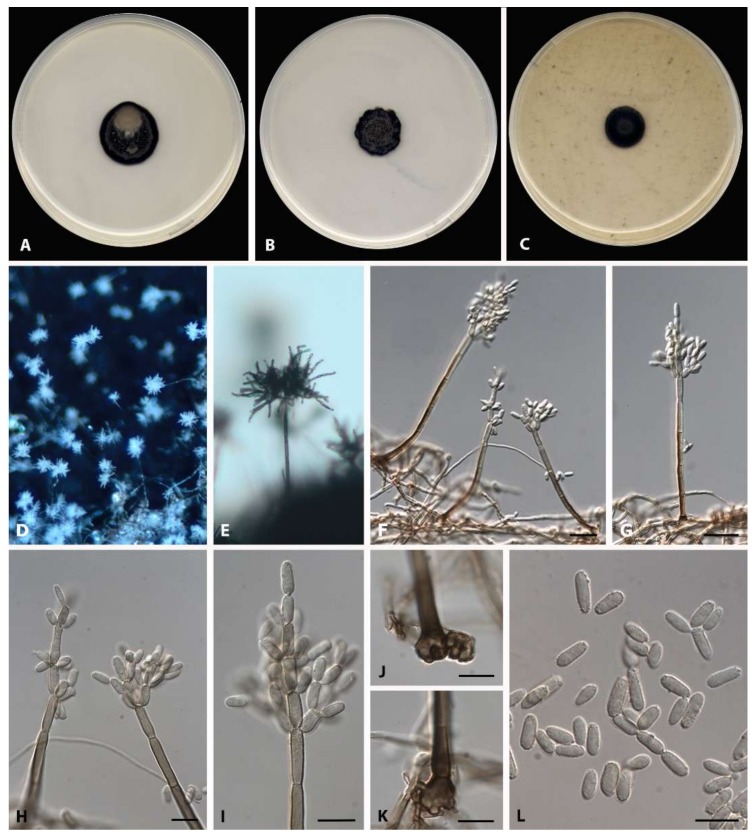
*P. vietnamensis* (ex-type FMR 17593). (**A**–**C**) Colonies on PDA, PCA, and OA, respectively, after 30 days at 25 °C. (**D**–**I**,**L**) Conidiophores and conidia. (**D**,**E**) Conidiophores under stereomicroscope. (**J**,**K**) Detail of conidiophore basal cells. Scale bars: (**F**,**G**) = 20 µm; (**H–L**) = 10 µm.

*Pseudopenidiella vietnamensis* Iturrieta-González, Dania García, Gené, sp. nov.—MycoBank MB 833180 (Figure 3).

Etymology: Name refers to Vietnam, the country where the fungus was collected.

Mycelium consisting of branched, septate, pale brown, smooth-walled hyphae 1–3 µm wide. Conidiophores macronematous, mononematous, unbranched, erect to slightly flexuous, subcylindrical, with up to 10-septate, pale brown to brown, smooth-walled, verruculose towards the apex, swollen and often lobate basal cell, up to 236 µm long, 3–5 µm wide; microconidiophores not observed. Conidiogenous cells terminal, polyblastic, with up to three inconspicuous conidiogenous loci, verruculose, pale brown, 12–18 × 2–4 µm. Ramoconidia cylindrical, aseptate, pale brown, verruculose, 7–13 × 3–4 µm, forming conidia in acropetal branched chains. Conidia cylindrical, aseptate, pale brown, smooth-walled to verruculose, 5–10 × 2–3 µm. Sexual morph not observed.

Culture characteristics after 30 days at 25 °C: Colonies on PDA reaching 23 mm of diameter, grey (7F1) with some regions greyish brown (5D3), black at the edge, velvety, slightly convex, aerial mycelium scarce, margin entire; reverse dark brown (7F8) to black. On PCA, reaching 17 mm of diameter, brownish grey (5F2), velvety, slightly convex, aerial mycelium scarce, margin undulate; reverse dark brown (7F8) to black. On OA, reaching 15 mm of diameter, brownish grey (5F2), black at the edge, finely granular, flat, aerial mycelium scarce, margin entire; reverse dark brown (7F6) to black.

Cardinal temperatures for growth: Minimum 15 °C, optimum 25 °C, maximum 30 °C.

Specimen examined: Vietnam, north-east region, on unidentified dead leaf, Aug. 2011, Josep Guarro (holotype CBS H-24292, culture ex-type CBS 146219 = FMR 17593). 

Diagnosis: *P. vietnamensis* differs from *P. piceae* and *P. gallaica* in the lack of microconidiophores, and from *P. podocarpi* in the size of their macronematous conidiophores (Table 3).

## 4. Discussion

Sequence analysis of the ITS and LSU barcodes were enough to resolve the taxonomy of the fungi under study and attribute the species to the monophyletic genera *Heliocephala* and *Pseudopenidiella*. However, phylogenetic relationships to other genera in the family *Microthyriaceae* remained obscure with the present taxon sampling, due to the lack of statistical support in the main clades obtained in the analysis (Figure 1). Despite DNA data not being available for all species of the mentioned genera, the novel species, *H. variabilis* and *P. vietnamensis,* showed morphological traits that clearly allowed their distinction from the other species of the respective genera (Table 2 and Table 3).

*Heliocephala variabilis* was phylogenetically close to *H. elegans, H. gracilis*, and *H. zimbabweensis.* The first two species, which were previously included in the genus *Holubovaniella* [10], could be differentiated from *H. variabilis* by having much more robust conidiophores (up to 700 × 11 µm in *H. elegans*; up to 350 × 10 µm in *H. gracilis*) that usually proliferate, showing several clusters of short branches and intercalary conidiogenous cells. In addition, on the natural substratum, our species showed conidia with up to four septa, while those of *H. elegans* and *H. gracilis* are 1–3- and 0–1-septate, respectively [10]. *Heliocephala zimbabweensis* resembles *H. proliferans*, and both differ from *H. variabilis* by having longer conidiophores (up to 210 µm in *H. proliferans*; up to 240 µm in *H. zimbabweensis*) and two-septate conidia with a very long and filiform rostrum, subsequently showing much longer conidia (10–200 µm in *H. proliferans*; 23–125 µm in *H. zimbabweensis*) than the species proposed here. Another feature exclusive to *H. proliferans* and *H. zimbabweensis* is the presence of a secondary cluster of conidiogenous cells at the apex of the conidial rostrum [8,29]. The great morphological similarity of these two species suggest they could be conspecific, but the lack of DNA data from the type is a handicap to elucidating the taxonomy of these fungi. Other two *Heliocephala* species with no molecular data are *Heliocephala triseptata* and *Heliocephala vietnamensis* [9,28], but the protologue of both taxa only describes conidia with three septa, and the conidiophores of the former are the smallest ones in the genus (21–40 µm long), while those of *H. vietnamensis* are longer (up to 340 µm) than those observed in *H. variabilis* (up to 153 µm long).

Although the three species of *Pseudopenidiella* are phylogenetically well differentiated, morphologically they are very similar, and even their conidiogenous apparatus resembles that of other cladosporium-like fungi that belong to the order *Capnodiales,* such as *Penidiella* or *Apenidiella* and other related genera [30,31]. *Pseudopenidiella* species can be only distinguished by subtle differences in their macroconidiophores and by the presence or absence of microconidiophores, as summarized in Table 3. It is worth mentioning that, based exclusively on morphological data, a fourth species named *Pseudopenidiella pini* was introduced in the genus by Kirk [32]. This was based on *Polyscytalum pini*, which was described from several specimens collected on decaying needles of *Pinus sylvestry,* mainly in the United Kingdom. However, none of these specimens is currently available for molecular comparison. Some of the features described and illustrated in its protologue, such as the presence of denticulate conidiogenous cells and one-septate (ramo-) conidia [33], do not fit with the generic concept of *Pseudopenidiella* [12]; therefore, we prefer to exclude *P. pini* from the genus until further studies with additional new collections of the species can confirm its position.

## Figures and Tables

**Table 1:** Species included in this study, their substrate, origin, and GenBank accession numbers.

Species	Strain Number ^1^	Substrate	Country	Genbank Accession No. ^2^	
				ITS	LSU
*Chaetothyriothecium elegans*	CPC 21375 ^T^	Leaves of *Castanopsis* sp.	Thailand	-	KF268420
*Heliocephala elegans*	MUCL 39003	Fallen leaf of *Andira inermis*	Cuba	HQ333478	HQ333478
*H. gracilis*	CBS 369.86 ^IT^	Fallen leaf of *Matayba oppositifolia*	Cuba	HQ333479	HQ333479
*H. natarajanii*	MUCL 43745 ^T^	Basideocarp of *Pisolithus tinctorius*	India	HQ333480	HQ333480
*H. zimbabweensis*	CBS 691.97 ^T^	Unidentified leaf litter	Zimbabwe	HQ333481	HQ333481
*H. variabilis* sp. nov.	FMR 17592 ^T^	Unidentified dead leaves	Vietnam	**LR536989**	**LR588212**
*Microthyrium buxicola*	MFLUCC 15-0212 ^T^	Leaves of *Buxus* sp.	Italy	-	KT306551
	MFLUCC 15-0213	Leaves of *Buxus* sp.	Italy	-	KT306552
*M. microscopicum*	CBS 115976 ^T^	-	The Netherlands	-	GU301846
*M. propagulensis*	IFRD 9037 ^T^	Fallen leaves of *Castanopsis histrix*	China	-	KU948989
*Pseudopenidiella piceae*	CBS 131453 ^T^	Needle litter of *Picea abies*	Czech Republic	JX069868	JX069852
*P. gallaica*	CBS 121796 ^T^	Unidentified dead leaves	Spain	LT984842	LT984843
*P. podocarpi*	CBS 146067 ^T^	Leaves of *Podocarpus latifolius*	South Africa	MN562140	MN567647
	CPC 37094	Leaves of *P. latifolius*	South Africa	MN562141	MN567648
*P. vietnamensis* sp. nov.	FMR 17593 ^T^	Unidentified dead leaves	Vietnam	**LR536990**	**LR536991**
*Stomiopeltis betulae*	CBS 114420	*Betula* sp.	Sweden	GU214701	GU214701
*Tumidispora shoreae*	MFLUCC 12-0409 ^T^	Dead leaves of *Shorea* sp.	Thailand	-	KT314073
	MFLUCC 14-0574	Dead leaves of *Shorea* sp.	Thailand	-	KT314074
*Venturia catenospora*	CBS 447.91 ^T^	Leaf spot of *Salix triandra*	Germany	EU035427	MH873940
*V. inaequalis*	CBS 476.61	Fruit of *Malus sylvestris*	Belgium	EU282478	GU456336

**^1^** CBS: culture collection of the Westerdijk Fungal Biodiversity Institute, Utrecht, The Netherlands; CPC: culture collection of Pedro Crous housed at the CBS; IFRD: International Fungal Research and Development Centre Research Institute of Resource Insects, Kunming; FMR: Facultat de Medicina, Universitat Rovira i Virgili, Reus, Spain; MFLUCC: Mae Fah Luang University Culture Collection, Chiang Ria, Thailand; MUCL: Mycothèque de L’Université Catholique de Louvain, Louvain-la-Neuve, Belgium; T and IT: ex-type and ex-isotype strain, respectively. **^2^** Sequences newly generated in this study are indicated in bold.

**Table 2:** Key morphological features distinguishing the accepted *Heliocephala* species.

Species	Conidiophore Size *	Conidia				References
		Size *	Septum No.	Ornamentation	Rostrum	
*H. elegans*	250–700 × 7–11	8–25 × 3–4	1–3	Smooth	Present, straight	[10]
*H. gracilis*	80–350 × 7–10	4–12.5 × 2–5	0–1	Smooth	Absent	[10]
*H. natarajanii*	up to 109 × 1.5–3.5	(8.5–)17–34(–103) × (1.5–) 2.5–4.5(–6.5)	2(–3)	Basal cell verruculose	Present, straight, curved or uncinate	[11]
*H. proliferans*	up to 210 × 3.5–4	(10–)15–50(–200) × 3–4	2	Basal cell verruculose	Present, straight or curved	[8]
*H. triseptata*	21–40 × 7–19	15–27 × 3.5–4.5	3	Smooth	Present, straight	[9]
*H. variabilis*	up to 153 × 4–6	4–26 × 3–6	(0–)1–3(–4)	Smooth to verruculose	Absent	Present study
*H. vietnamensis*	210–340 × 6–8	14–17 × 2.8–3.8	3	Smooth	Absent	[28]
*H. zimbabweensis*	180–240 × 3–4	23–125 × 3.5–5.3	2	Smooth	Present, straight	[29]

* in μm.

**Table 3:** Key morphological features distinguishing the accepted *Pseudopenidiella* species.

Species	Macroconidiophore Size *	Microconidiophore	Ramoconidia Size *	Conidia		References
				Size *	Ornamentation	
*P. gallaica*	up to 120 × 2–3	Present	7.5–11 × 2–3	6–12 × 1–3	Smooth to verruculose	[7]
*P. piceae*	up to 150 x 3–4	Present	8–12 × 2–3	(6–)7–9(–10) × (2.5–)3	Finely verruculose	[12]
*P. podocarpi*	10–110 × 3–4	Absent	(9–)12–13 × (2.5–)3–3.5	(9–)11–12(–15) × 2.5(–3)	Verruculose	[13]
*P. vietnamensis*	up to 236 × 3–5	Absent	7–13 × 3–4	5–10 × 2–3	Smooth	Present study

* in μm.
